# Possible roles of sestrin2 in multiple sclerosis and its relationships with clinical outcomes

**DOI:** 10.1590/0004-282X-ANP-2021-0202

**Published:** 2022-02-21

**Authors:** Faruk Omer ODABAS, Ali Ulvi UCA, Turan AKDAG, Filiz DEMİRDÖGEN, Mustafa ALTAS, Osman Serhat TOKGOZ

**Affiliations:** 1 University of Health Sciences, Konya City Hospital, Department of Neurology, Konya, Turkey.; 2 Necmettin Erbakan University, Meram Medical School, Department of Neurology, Konya, Turkey.; 3 Necmettin Erbakan University, Vocational School of Meram, Konya, Turkey.; 4 Binali Yıldırım Unıversıty Mengücek Gazi Educatıon and Research Hospıtal, Erzincan, Department of Neurology, Turkey.

**Keywords:** Multiple Sclerosis, Sestrins, Apoptosis, Biomarkers, Inflammation, Oxidative Stress, Esclerose Múltipla, Sestrinas, Apoptose, Biomarcadores, Inflamação, Estresse Oxidativo

## Abstract

**Background:**

Characterized by demyelination, inflammation and axonal damage, multiple sclerosis (MS) is one of the most common disorders of central nervous system led by the immune system. There is an urgent and obvious need for biomarkers for the diagnosis and follow-up of MS.

**Objective:**

To investigate serum levels of sestrin2 (SESN2), a protein that responds to acute stress, in MS patients.

**Methods:**

A total of 85 participants, 40 patients diagnosed previously with relapsing-remitting MS and 45 healthy controls, were included. Serum SESN2 parameters were investigated in blood samples drawn from each participant in the patient and control groups.

**Results:**

SESN2 levels were significantly lower in MS patients than in controls (z: -3.06; p=0.002). In the ROC analysis of SESN2, the predictive level for MS was 2.36 ng/mL [sensitivity, 72.50%; specificity, 55.56%; p=0.002; area under the curve (AUC)=0.693]. For the cut-off value in both groups, SESN2 was an independent predictor for MS [Exp (B)=3.977, 95% confidence interval (95%CI) 1.507-10.494 and p=0.013].

**Conclusions:**

The decreased expression of SESN2 may play a role in MS pathogenesis, and SESN2 could be used as a biomarker for MS and as immunotherapeutic agent to treat MS.

## RESUMO

**Antecedentes:**

Caracterizada por desmielinização, inflamação e dano axonal, a esclerose múltipla (EM) é uma das doenças mais comuns do sistema nervoso central liderada pelo sistema imunológico. Há uma necessidade urgente e óbvia de biomarcadores para o diagnóstico e acompanhamento da EM.

**Objetivo:**

Investigar os níveis séricos de sestrina2 (SESN2), uma proteína que responde ao estresse agudo, em pacientes com EM.

**Métodos:**

Foram incluídos 85 participantes, 40 pacientes com diagnóstico prévio de EM recorrente-remitente e 45 controles saudáveis. Os parâmetros do SESN2 sérico foram investigados em amostras de sangue coletadas de cada participante nos grupos de paciente e controle.

**Resultados:**

os níveis de SESN2 foram significativamente mais baixos em pacientes com EM do que em controles (z: -3,06; p=0,002). Na análise ROC do SESN2, o nível preditivo para MS foi 2,36 ng/mL [sensibilidade, 72,50%; especificidade, 55,56%; p=0,002; área sob a curva (AUC)=0,693]. Para o valor de corte em ambos os grupos, SESN2 foi um preditor independente para MS [Exp (B)=3,977, intervalo de confiança de 95% (95%CI) 1,507-10,494; p=0,013].

**Conclusões:**

A expressão diminuída de SESN2 pode desempenhar um papel na patogênese da EM, e SESN2 poderia ser usado como um biomarcador para EM e como agente imunoterapêutico para o tratamento de EM.

## INTRODUCTION

Multiple sclerosis (MS) is one of the most common diseases of the central nervous system (CNS) led by the immune system and is known to cause demyelination, inflammation, and axonal damage^1^. Whether or not inflammation and neurodegeneration are causally associated with MS remains unclear. The sequence of a potential causal correlation is also unknown. The observations obtained in most experimental studies seem to support a pathogenesis in which the inflammation precedes neurodegeneration^2^. The accumulation of inflammatory cells in the CNS is a critical step in the development of demyelination in MS. The migration of inflammatory cells into the CNS may occur through the synthesis of members of many chemokine families in CNS^3^. In addition, the activation of myelin-specific T cells can cross the blood-brain barrier, and the proliferation of these cells occurs. After proliferation, myelin-specific T cells release proinflammatory cytokines, which in turn stimulate microglia, macrophages, and astrocytes^4^.

The diagnosis of MS is currently based on clinical evaluations. Molecular biomarkers of MS have been mainly restricted to measurement in cerebrospinal fluid. Although the clinical utility of conventional magnetic resonance imaging (MRI) in diagnosis and treatment of MS is clear in daily practice, MRI has numerous limitations^5^. In recent studies, it was revealed that MS is a commonly misdiagnosed disorder, even among scholars with expertise^6^. There is an urgent and obvious need for improved methods to diagnose MS and follow-up the prognosis. New approaches to improving diagnostic accuracy of MS could prevent the unnecessary risks and morbidity associated with misdiagnosis, as well as the disabilities that will be experienced by MS patients^5^.

In recent studies, it has been shown that newly identified cytokines and proteins can make important clinical contributions to the diagnosis and treatment of diseases. Nowadays, the roles of sestrin molecules (SESNs) have been well-established in various disorders, including neurological diseases. Sestrin2 (SESN2) is an important member of the SESN family (SESN1, SESN2 and SESN3), a set of highly conserved proteins induced by environmental stresses such as DNA damage, inflammation, autophagy, oxidative stress, and hypoxia^7^ ^,^ ^8^ ^,^ ^9^. SESN2 has also been shown to be responsible for free radicals scavenging and autophagy, which initiate cell protection activities^8^. Additionally, SESN2 is crucial for antioxidant defense through the regeneration of peroxiredoxins by regulating the adenosine monophosphate-activated protein kinase (AMPK)/mammalian target of rapamycin (mTOR) pathway, thereby controlling cell growth and metabolism^10^. Developing sensitive and specific biomarkers to accurately differentiate MS from other disorders still remains a pressing and unmet need in the field. Although the association between SESN2 and several other neurological diseases has been investigated in various studies^11^ ^,^ ^12^, there are no data related to the connection between SESN2 and MS. The aim of this study was to contribute to the literature by assessing SESN2 levels to determine if SESN2 may be used as a biomarker for MS and to evaluate its relationship to clinical outcomes.

## METHODS

This study was approved by the ethics committee of our institution, and a written informed consent was obtained from all participants. The present study was conducted under the Good Clinical Practice guidelines of the Declaration of Helsinki and its later amendments.

### Participants

MS patients admitted to the outpatient clinic for control purposes between June 2020 and March 2021 constituted the study population. Forty individuals with relapsing-remitting multiple sclerosis (RRMS) were consecutively defined to be included and excluded from the study. The controls were composed of 45 healthy volunteers having no known medical disorders and matched in terms of age and gender. The 40 patients in the MS group were receiving disease modifying therapy (DMT) including interferon beta-1a (8 patients), interferon beta-1b (5 patients), glatiramer acetate (6 patients), teriflunomide (5 patients), dimethyl fumarate (3 patients), and fingolimod (13 patients).

Inclusion criteria for the patient group were: voluntary participation; individuals aged 18 to 55 years; individuals meeting the McDonald’s criteria for RRMS diagnosis in terms of time and space dissemination according to the 2010 version; individuals with an Expanded Disability Status Scale (EDSS) score below 5.5; and individuals with no acute or chronic disease detected other than MS.

The exclusion criteria for the patient group were: individuals with diagnosis of radiologically isolated syndrome, clinically isolated syndrome, primary/secondary MS, and RRMS who had an attack in the past 3 months; patients with a history of drug or substance addiction/abuse; and patients who were using oral or pulse corticosteroids, anticoagulants, selective serotonin reuptake inhibitors, and antipsychotic drugs.

### Measurement of sestrin2

Blood samples drawn from each participant within 30 minutes’ time were centrifuged at 3000 rpm for 15 minutes, and then the obtained sera were kept at -80°C until analysis. Serum levels of SESN2 were determined by the enzyme-linked immunosorbent assay (ELISA) technique. The serum concentrations of SESN2 were analyzed by Human SESN2 ELISA kits (Bioassay Technology Laboratory, Shanghai, China; catalog number, E3437Hu). The sensitivity was 0.01 ng/mL and the standard curve range was 0.05-15 ng/mL, with intra- and inter-assays of <8 and <10%, respectively. The manufacturer’s instructions were followed. The absorbances of the specimens were measured at 450 nm using the absorbance microtiter plate reader with a double-blind procedure (ELx800^TM^, Bio-Tech Instruments, USA).

### Statistical analysis

The statistical analyses were conducted using the *Standard Package for the Social Sciences* for Windows, version 15.0 (SPSS, Chicago, IL, USA). Data are reported as mean values and standard deviations (±SD) or medians and percentiles with a 25-75% quartiles. The Kolmogorov Smirnov test was used for normally distributed variables. For parametric comparisons between the two groups, the Student’s *t*-test was used, while the Mann-Whitney U test was used for nonparametric comparisons. The chi-square test was also used for the comparison of the categorical data.

The receiver operating characteristic (ROC) was used to analyze the areas under the curve (AUC), sensitivity, specificity, and positive and negative predictive values. In addition, the binary logistic regression analysis was performed to determine the independent predictive risk factors for MS. P values less than 0.05 were accepted as statistically significant.

## RESULTS

Eighty-five volunteers (40 in the patient group and 45 in the control group) with a mean age of 38.22±8.75 were included in the study. The demographic and clinical characteristics of the patients and controls are shown in Table 1.

**Table 1.** The demographic and clinical characteristics of patients and controls.

	RRMS			
	Patients (n=40)		Healthy controls (n=45)	
	Mean	Quartile (25-75%)	Mean	Quartile (25-75%)
Age (years) (mean±SD)	-	38.7 (±8.6)	-	37.6 (±8.9)
Sex (female)	25 (62.5%)	-	26 (57.8%)	-
Disease duration (years)	7.5	5.0-12.0	-	-
Number of MS attacks	4.0	2.0-5.0	-	-
EDSS	1.5	1.0-2.0	-	-
SESN2 (ng/mL)	1.64	0.91-2.47	2.54	1.36-9.52
	DMT use	DMT use duration (years-mean)		
Interferon beta-1a, n (%)	8 (20%)	3.6	-	-
Interferon beta-1b, n (%)	5 (12.5%)	3.8	-	-
Glatiramer acetate, n (%)	6 (15%)	3.5	-	-
Teriflunomide, n (%)	5 (12.5%)	2.4	-	-
Dimethyl fumarate, n (%)	3 (7.5%)	2.3	-	-
Fingolimod, n (%)	13 (32.5%)	3.1	-	-

DMT: disease modifying therapy; EDSS: Expanded Disability Status Scale; MS: multiple sclerosis; RRMS: relapsing remitting multiple sclerosis; SD: standard deviation.

**Figure 1.**
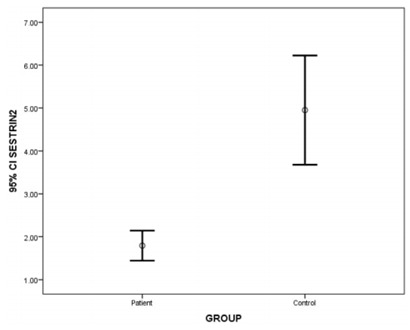
Mean levels of sestrin2 in patient and control groups.

No significant difference was detected between the levels of SESN2 in terms of gender (p=0.299). There was also no significant difference between levels of SESN2 and drug therapies used by MS patients (chi-square=4.608; p=0.595). Levels of SESN2 were significantly lower in patients with MS, compared with those in the controls (z=-3.06; p=0.002), and the findings are presented in Figure 1.

**Figure 2.**
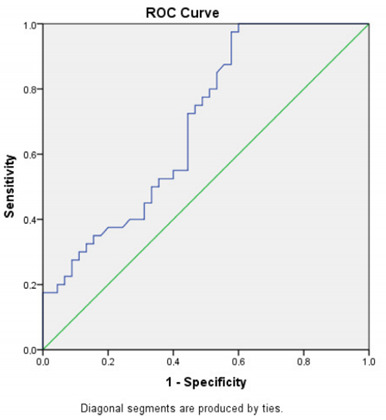
Receiving operating characteristics curve of sestrin2 for the prediction of multiple sclerosis in the patient group.

As shown in Figure 2, the predictive level of SESN2 for MS in ROC analysis was 2.36 ng/mL [sensitivity, 72.50%; specificity, 55.56%, positive predictive value (PPV), 59.18%; negative predictive value (NPV), 69.44%; p=0.002; and AUC=0.693 (0.582-0.804)]. The cut-off value of 2.36 ng/mL for SESN2 was the statistically significant explanatory variable for the dependent variables (p<0.001). Values lower than 2.36 ng/mL were seen 3.9 times more often in patients. The overall corrected percentage was 63.5% (Table 2).

**Table 2.** Binary logistic regression analysis for sestrin2.

	Exp (B) p-value	95%CI for Exp (B) p-value	Lower	Upper
Age	0.321	0.974	0.924	1.026
Sex	0.255	0.573	0.220	1.494
SESN2<2.36 (ng/mL)	0.013	3.977	1.507	10.494
Constant	0.409	2.461	-	-

R^2^: 13.6 %; 95%CI: 95% confidence interval; SESN2: sestrin2.

No correlation was found between levels of SESN2 and number of MS attacks (p>0.05) and between levels of SESN2 and age (p>0.05). In addition, no correlations were found between SESN2 levels and EDSS (p>0.05) and between SESN2 levels and disease duration (p>0.05). Likewise, no significant difference was found between various DMT regarding SESN2 levels (p>0.05).

## DISCUSSION

To the best of our knowledge, our study was the first to evaluate SESN2 levels in MS. In our study, the levels of serum SESN2 were found to be significantly decreased in the MS group compared with the controls. On the other hand, no correlation between SESN2 and age, sex, disease duration, clinical severity measured by EDSS, number of attacks and DMT was found. This might indicate that the molecular difference in SESN2 levels between both groups began probably in the early stages of the disease. Given the inflammatory nature of MS, it was intriguing to observe lower serum SESN2 levels in our MS patients. However, we believe that more comprehensive studies are needed to investigate the cause of such a situation.

Recent evidence has revealed that three different types of SESN are responsible for performing diverse functions. Specifically, SESNs have a protective effect on lymphocytes by diminishing reactive oxygen species (ROS) levels arising from oxidative and genotoxic stresses^13^. Of the three types of SESNs, SESN2 is the one that has been most extensively investigated in many studies since 2002, the year of its discovery^14^. The number of studies assessing the other types of SESNs is restricted. Recognized as a p53-activated gene 26 (PA26) due to its regulation by tumor-suppressor protein (p53), SESN1 has been accepted as one of the genes stopping tumor growth and leading to the impairment of DNA structure^13^. However, SESN2, a homolog of PA26, can also lead to hypoxia of gene 95 on account of its induction under hypoxic situations, although other cytotoxic events such as oxidative stress and DNA damage also induce SESN2^13^ ^,^ ^15^ ^,^ ^16^. SESN3 is also accepted as a new gene associated with PA26, led by the forkhead box O (FoxO) family of transcription factors^17^.

In many studies, SESN2 has been shown to have significant influences on immune cells. SESN2 is likely to play a part in innate and acquired cells of the immune system, such as monocytes, macrophages, natural killer, and T cells^18^ ^,^ ^19^. Various stress-originated problems elevate the level of SESN2 by regulating various crucial transcription factors. Processes such as the concentration of ROS, protein synthesis, lipogenesis, regeneration of cells and detrimental effects on DNA are suppressed by the upregulation of SESN2, which decreases the levels oxidative stress in endoplasmic reticulum (ER), activating autophagy or relieving inflammasome activation^14^ ^,^ ^18^ ^,^ ^20^ ^,^ ^21^ ^,^ ^22^. Through these regulatory roles, SESN2 could be used in the treatment of some inflammatory diseases^7^.

Some studies have reported that SESNs are of a vital role in various disorders, including neurological diseases^7^ ^,^ ^11^ ^,^ ^12^. The levels of SESN2 were found to be increased in individuals with various diseases, and the plasma levels were stated to have positive effects in decreasing disease severity^23^ ^,^ ^24^. Sepsis, liver diseases, ischemia-reperfusion (I/R) injury (myocardial and cerebral I/R injury), cardiovascular diseases such as chronic heart failure, coronary artery diseases, aortic dissection and atrial fibrillation, chronic obstructive pulmonary disease, metabolism-related diseases including diabetes mellitus, obesity, cancer and aging are among the disorders influenced by SESN2^11^. The effects of SESNs on neurological ailments have yet to be precisely revealed. However, SESNs have drawn increasing attention in seizures, neuropathy-related pain, ischemic stroke, Alzheimer’s disease (AD), Parkinson’s disease (PD), Huntington’s disease, and amyotrophic lateral sclerosis^7^ ^,^ ^11^ ^,^ ^12^. Excessive oxidative stress and autophagy have important effects on the pathogenesis of neurological diseases related to advanced age, especially degenerative disorders^7^ ^,^ ^12^. For example, an upregulated serum SESN2 level was observed in PD group compared to control group^25^. Another study showed significant overexpression of serum SESN2 protein and mRNA levels in the AD group compared to mild cognitive impairment patients and elderly control groups^26^. SESN2-knockdown was also shown to strongly increase lipopolysaccharide (LPS)-mediated nuclear factor-κB phosphorylation by decreasing AMPK phosphorylation and thus leading to the up-regulation of several adhesion molecules in the endothelium and expression of proinflammatory cytokines^27^. As a result, SESN2-knockdown increased the production of LPS-induced ROS, ER stress, and cell death. In several studies, it was shown that SESN2 inhibits the inflammatory pathway and decreases the extent of inflammation in macrophages, which is a significant mediator for the formation of atherosclerosis^19^ ^,^ ^28^ ^,^ ^29^.

Through the genetic deletion of SESNs in animal models, especially mice, valuable information has been revealed on the vital effects of such proteins. Deprived of three types of SESNs, mice had reduced rates of postnatal survival associated with defective mTORC1 inactivation in multiple organs during neonatal fasting. In these animals, a non-redundant mechanism has been revealed, by which the sestrin family of guanine nucleotide dissociation inhibitors regulates the nutrient-sensing Rag GTPases to control the signals of mTORC1^30^. SESN2-knocked-out mice have shown proliferation of pro-inflammation genes and the activation of basilar membrane macrophages. Based on these results, SESN2 is suggested to have significant effects on cochlear homeostasis and immune responses as components of stress^31^. Other phenotypes of SESN2-knocked out mice involved the impaired hair cells in cochlear explants administered with gentamicin. In this trial, mice also displayed elevated neuropathy-related pain due to increased ROS levels in the late phase^32^. The loss of SESN2 activity is likely to contribute to the cellular accumulation of ROS, which can promote DNA damage and genomic mutations facilitating the development of tumors^33^. In previous studies, the down-regulation of SESN2 was shown to accelerate both colitis and colon carcinogenesis, while SESN1 and SESN3 were found to be strongly down-regulated in various types of cancer tissues, such as lung cancers and lymphomas^34^.

The specific elements causing the pathogenesis of MS remain unknown. Recent evidence has suggested that inflammation, apoptosis, and oxidative/nitroxidative stress are important contributors to etiology, progression and clinical symptoms of MS^1^. In our study, values below the cut off value of 2.36 ng/mL for SESN2 was observed at a higher rate among MS patients (3,977 times higher), compared to the controls. In other words, significantly down-regulated levels of serum SESN2 were observed in patients with MS compared to controls. The data obtained in our study indicate that SESN2 levels were able to differentiate between patient and control groups. The above information in the literature suggests that lower levels of SESN2 may play a very important role in the development of MS by triggering inflammatory processes. In addition, the lack of a statistical significance between levels of SESN2 and age of MS patients suggests that low levels of SESN2 occur at the onset of MS. Future studies should determine the levels of SESN2 in individuals followed-up due to diagnosis of radiologically isolated syndrome (RIS) and confirm such a hypothesis. Studies on the up-regulation of SESN2 levels in MS patients and evaluating other members of the SESNs family in MS patients can help us to better understand the disease and develop treatment strategies. Therefore, we consider that SESN2 could have a significant effect as a biomarker of immunity in diagnosing MS and as an MS treatment.

However, our study has some limitations. First, the sample size was relatively small. Secondly, we evaluated only individuals with no MS attacks in the RRMS group. Thus, our results should be verified by further studies to be conducted in those with RIS, clinically isolated syndrome, progressive MS and MS attacks. Further studies with larger sample sizes, longitudinal evaluation and assessment of post-treatment levels will be more comprehensive in revealing the cause-effect relationship between SESN2 and MS.

In conclusion, we found that SESN2, an acute-stress responsive protein, was decreased in MS. Our findings also suggest that decreased SESN2 levels may cause demyelination and axonal damage in MS through inflammation, oxidative stress, and apoptosis. Our study might lead to further studies on this molecule and to the investigation of its use as a treatment option, as it is likely to prevent or slow down disease progression. SESN2 could play a part as a biomarker for MS diagnosis and as immunotherapy to treat MS.

## References

[1] Reich DS, Lucchinetti CF, Calabresi PA (2018). Multiple Sclerosis. N Engl J Med.

[2] Milo R, Korczyn AD, Manouchehri N, Stüve O (2020). The temporal and causal relationship between inflammation and neurodegeneration in multiple sclerosis. Mult Scler.

[3] Compston A (2004). The pathogenesis and basis for treatment in multiple sclerosis. Clin Neurol Neurosurg.

[4] Peterson LK, Fujinami RS (2007). Inflammation, demyelination, neurodegeneration and neuroprotection in the pathogenesis of multiple sclerosis. J Neuroimmunol.

[5] Oh J, Sicotte NL (2020). New imaging approaches for precision diagnosis and disease staging of MS?. Mult Scler.

[6] Kaisey M, Solomon AJ, Luu M, Giesser BS, Sicotte NL (2019). Incidence of multiple sclerosis misdiagnosis in referrals to two academic centers. Mult Scler Relat Disord.

[7] Pasha M, Eid AH, Eid AA, Gorin Y, Munusamy S (2017). Sestrin2 as a novel biomarker and therapeutic target for various diseases. Oxid Med Cell Longev.

[8] Lee JH, Budanov AV, Karin M (2013). Sestrins orchestrate cellular metabolism to attenuate aging. Cell Metab.

[9] Li L, Xiao L, Hou Y, He Q, Zhu J, Li Y (2016). Sestrin2 silencing exacerbates cerebral ischemia/reperfusion injury by decreasing mitochondrial biogenesis through the AMPK/PGC-1α Pathway in Rats. Sci Rep.

[10] Shi X, Xu L, Doycheva DM, Tang J, Yan M, Zhang JH (2017). Sestrin2, as a negative feedback regulator of mTOR, provides neuroprotection by activation AMPK phosphorylation in neonatal hypoxic-ischemic encephalopathy in rat pups. J Cereb Blood Flow Metab.

[11] Chen SD, Yang JL, Lin TK, Yang DI (2019). Emerging roles of sestrins in neurodegenerative diseases: counteracting oxidative stress and beyond. J Clin Med.

[12] Wang LX, Zhu XM, Yao YM (2019). Sestrin2: its potential role and regulatory mechanism in host immune response in diseases. Front Immunol.

[13] Budanov AV (2011). Stress-responsive sestrins link p53 with redox regulation and mammalian target of rapamycin signaling. Antioxid Redox Signal.

[14] Budanov AV, Shoshani T, Faerman A, Zelin E, Kamer I, Kalinski H (2002). Identification of a novel stress-responsive gene Hi95 involved in regulation of cell viability. Oncogene.

[15] Seo K, Seo S, Ki SH, Shin SM (2016). Sestrin2 inhibits hypoxia-inducible factor-1alpha accumulation via AMPK-mediated prolyl hydroxylase regulation. Free Radic Biol Med.

[16] Budanov AV, Sablina AA, Feinstein E, Koonin EV, Chumakov PM (2004). Regeneration of peroxiredoxins by p53-regulated sestrins, homologs of bacterial AhpD. Science.

[17] Chen CC, Jeon SM, Bhaskar PT, Nogueira V, Sundararajan D, Tonic I (2010). FoxOs inhibit mTORC1 and activate Akt by inducing the expression of Sestrin3 and Rictor. Dev Cell.

[18] Kim MJ, Bae SH, Ryu JC, Kwon Y, Oh JH, Kwon J (2016). SESN2/sestrin2 suppresses sepsis by inducing mitophagy and inhibiting NLRP3 activation in macrophages. Autophagy.

[19] Yang JH, Kim KM, Kim MG, Seo KH, Han JY, Ka SO (2015). Role of sestrin2 in the regulation of proinflammatory signaling in macrophages. Free Radic Biol Med.

[20] Budanov AV, Karin M (2008). p53 target genes sestrin1 and sestrin2 connect genotoxic stress and mTOR signaling. Cell.

[21] Jegal KH, Ko HL, Park SM, Byun SH, Kang KW, Cho IJ (2016). Eupatilin induces Sestrin2-dependent autophagy to prevent oxidative stress. Apoptosis.

[22] Saveljeva S, Cleary P, Mnich K, Ayo A, Pakos-Zebrucka K, Patterson JB (2016). Endoplasmic reticulum stress-mediated induction of sestrin2 potentiates cell survival. Oncotarget.

[23] Xiao T, Zhang L, Huang Y, Shi Y, Wang J, Ji Q (2019). Sestrin2 increases in aortas and plasma from aortic dissection patients and alleviates angiotensin II-induced smooth muscle cell apoptosis via the Nrf2 pathway. Life Sci.

[24] Ye J, Wang M, Xu Y, Liu J, Jiang H, Wang Z (2017). Sestrins increase in patients with coronary artery disease and associate with the severity of coronary stenosis. Clin Chim Acta.

[25] Rai N, Upadhyay AD, Goyal V, Dwivedi S, Dey AB, Dey S (2020). Sestrin2 as serum protein marker and potential therapeutic target for Parkinson’s disease. J Gerontol A Biol Sci Med Sci.

[26] Rai N, Kumar R, Desal GR, Venugopalan G, Shekhar S, Chatterjee P (2016). Relative alterations in blood-based levels of sestrin in Alzheimer’s disease and mild cognitive impairment patients. J Alzheimers Dis.

[27] Hwang HJ, Jung TW, Choi JH, Lee HJ, Chung HS, Seo JA (2017). Knockdown of sestrin2 increases pro-inflammatory reactions and ER stress in the endothelium via an AMPK dependent mechanism. Biochim Biophys Acta Mol Basis Dis.

[28] Yang K, Xu C, Zhang Y, He S, Li D (2017). Sestrin2 Suppresses Classically Activated Macrophages-Mediated Inflammatory Response in Myocardial Infarction through Inhibition of mTORC1 Signaling. Front Immunol.

[29] Kim MG, Yang JH, Kim KM, Jang CH, Jung JY, Cho IJ (2015). Regulation of Toll-like receptor-mediated Sestrin2 induction by AP-1, Nrf2, and the ubiquitin-proteasome system in macrophages. Toxicol Sci.

[30] Peng M, Yin N, Li MO (2014). Sestrins function as guanine nucleotide dissociation inhibitors for Rag GTPases to control mTORC1 signaling. Cell.

[31] Zhang C, Sun W, Li J, Xiong B, Frye MD, Ding D (2017). Loss of sestrin 2 potentiates the early onset of age-related sensory cell degeneration in the cochlea. Neuroscience.

[32] Kallenborn-Gerhardt W, Lu R, Syhr KM, Heidler J, von Melchner H, Geisslinger G (2013). Antioxidant activity of sestrin 2 controls neuropathic pain after peripheral nerve injury. Antioxid Redox Signal.

[33] Sablina AA, Budanov AV, Ilyinskaya GV, Agapova LS, Kravchenko JE, Chumakov PM (2005). The antioxidant function of the p53 tumor suppressor. Nat Med.

[34] Ro SH, Xue X, Ramakrishnan SK, Cho CS, Namkoong S, Jang I (2016). Tumor suppressive role of sestrin2 during colitis and colon carcinogenesis. Elife.

